# Unveiling the Synergistic Effect of Salicylic Acid on Triterpenoid Biosynthesis in *Athelia termitophila*: Elucidating the Molecular Underpinnings

**DOI:** 10.3390/ijms26030996

**Published:** 2025-01-24

**Authors:** Fangcheng Hu, Yonggang Fang, Zahid Khan, Lianxi Xing

**Affiliations:** 1 College of Life Sciences, Northwest University, Xi’an 710069, China; fangchengwho@163.com (F.H.); yahoofangyonggang@163.com (Y.F.); khanzahid370@yahoo.com (Z.K.); 2 Department of Zoology, University of Swabi, Swabi 23561, Pakistan; 3 Shaanxi Key Laboratory for Animal Conservation, Northwest University, Xi’an 710069, China; 4 Key Laboratory of Resource Biology and Biotechnology in Western China, Northwest University, Ministry of Education, Xi’an 710069, China

**Keywords:** *Athelia termitophila*, triterpenoids, salicylic acid, biotechnological source, molecular mechanism

## Abstract

This study investigates the dual role of salicylic acid (SA) in enhancing the production of triterpenes and elucidates its molecular regulatory mechanisms in the fungus *Athelia termitophila* (TMB), a rich source of bioactive triterpenoids vital to the cosmetics and pharmaceutical industries. Our innovative approach involves the strategic application of SA during the mycelial growth phase, leading to a remarkable 21.87% increase in triterpene yield under optimized conditions of 200 μmol/L SA over 9 days. Pioneering in its methodology, our research employs Spearman correlation analysis to dissect the intricate relationship between triterpene content and gene expression within the mevalonate (MVA) pathway of *A. termitophila*. This analysis has identified four key genes—Acetyl-Coa Acetyltransferase (*AACT)*, Squalene Epoxidase (*SE*), Phosphomevalonate Kinase (*PMK)*, and Mevalonate Diphosphate Decarboxylase *(MVD)*—that are important for triterpene synthesis, providing new insights into the biosynthetic capabilities of *A. termitophila*. Furthermore, our application of cluster analysis has unveiled unprecedented expression patterns among critical genes, at specific growth intervals. This novel insight into the temporal dynamics of gene transcription during triterpene synthesis provides a comprehensive view of the biosynthetic process, setting the stage for targeted enhancement of triterpene production in *A. termitophila*. This investigation not only highlights TMB’s potential as a biotechnological source of triterpenes but also provides critical insights into the underlying molecular pathways responsible for triterpene synthesis.

## 1. Introduction

There are many fungi that are symbiotic with termites, including *Termitomyces albuminosus* [1]. This termite symbiotic fungus is rich in essential amino acids, proteins, and other nutrients. In addition, its extract also has analgesic, anti-inflammatory, liver-protective, and in vitro antioxidant effects [2]. *Athelia termitophila* (*Atheliaceae: Basidiomycota*), a recently described fungus, has been identified in termite nests since its first discovery in 2006 [3,4]. We isolated a strain of *A*. *termitophila* (TMB) from a termite nest, and through molecular identification, confirmed it to be the same species described in Japan [5].

The mycelium of TMB has been verified to be nutrient-rich and safe through nutritional analysis and safety assessment, making it a potential raw material for food. Additionally, preliminary data suggest that the mycelium contains bioactive compounds—including triterpenes, fungal polysaccharides, cordycepin, and adenosine—that may have beneficial health effects. Its unique chemical composition and nutrient profile make TMB a valuable organism for studying bioactive compounds, particularly triterpenoids. Triterpenes, a class of terpenoids composed of 30 carbon atoms, have broad industrial applications in the food [6], cosmetics, and pharmaceutical industries due to their potent pharmacological properties, which include immunomodulatory, antioxidant, and anticancer qualities [7]. For example, triterpenes present in hydrolysates of total saponins have been shown to have potential applications in functional diets aimed at preventing myocardial damage and liver fibrosis [8]. The total triterpenes of *Ganoderma lucidum* have been utilized for centuries in traditional Chinese medicine for cancer prevention and treatment [9]. The triterpenes of the herbaceous plant *Centella asiatica* can effectively improve venous wall changes and protect the venous endothelium in chronic venous hypertension [10]. Likewise, apple-derived triterpenes are linked to the prevention of various chronic diseases, including cancer and cardiovascular conditions [11]. Despite the significant pharmaceutical potential of terpenoids, they remain underexplored.

Triterpenes are secondary metabolites that are typically present in low concentrations under natural conditions. Therefore, various methods need to be employed to increase their yield. The primary strategies for enhancing triterpene production involve optimizing their genetic metabolic pathways and employing exogenous compounds to stimulate triterpene biosynthesis [12]. Among these, the chemical induction of triterpene synthesis is the most economical and commonly used method. Salicylic acid (SA), an exogenous chemical inducer, plays a pivotal role as a signal transduction molecule in secondary metabolism [13]. Previous studies have shown that SA can induce the expression of key genes in the MVA pathway, thereby promoting triterpene accumulation [14,15].

**Figure 1:**
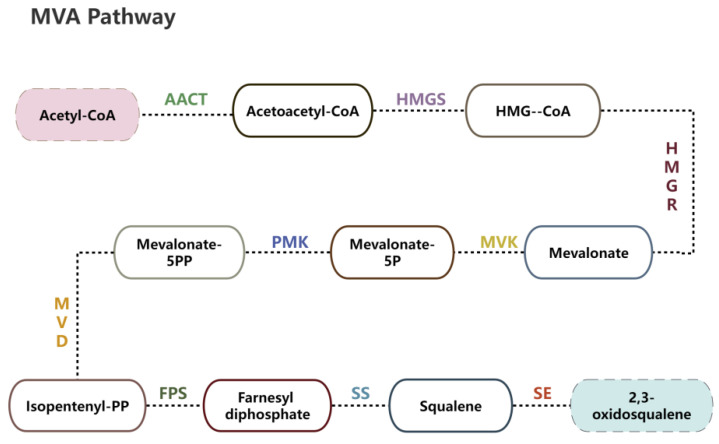
A schematic representation of mevalonate biosynthetic pathways for triterpenoids. AACT: Acetyl-Coa Acetyltransferase, HMGS: 3-Hydroxy-3-Methylglutaryl-Coa-Synthase, HMGR: 3-Hydroxy-3-Methylglutaryl-Coa Reductase, MVK: Mevalonate Kinase, PMK: Phosphomevalonate Kinase, MVD: Mevalonate Diphosphate Decarboxylase, FPS: Farnesyl Diphosphate Synthase, SS: Squalene Synthase, SE: Squalene Epoxidase.

The MVA pathway, a critical route for triterpenoid biosynthesis, involves several key genes, each catalyzing specific enzymatic steps (Figure 1). *AACT* initiates the pathway by catalyzing the condensation of acetyl-CoA. It is followed by *HMGS* and the rate-limiting enzyme *HMGR*, which facilitate the formation of mevalonate [16]. Further downstream, *PMK* phosphorylates mevalonate, and *MVD* subsequently catalyzes its decarboxylation to produce IPP, the essential precursor for triterpenoid synthesis [17]. *IDI* regulates the balance between IPP and DMAPP, ensuring a dynamic equilibrium of these critical intermediates. *FPS* then condenses IPP and DMAPP to form FPP, the direct precursor for squalene [18]. At the downstream stage of the pathway, *SS* catalyzes the conversion of FPP into squalene, and *SE* oxidizes squalene to 2,3-oxidosqualene [19], a key intermediate for triterpenoid formation. The coordinated expression of these genes directly regulates pathway flux and triterpenoid accumulation.

Currently, most research on TMB has focused on its symbiotic and parasitic relationships with termites [20,21]. However, little is known about the impact of SA on its metabolites and the key enzymes or genes involved in the synthetic pathway. This study has two main objectives: (1) to evaluate TMB as a potential fungal source of triterpenes under SA stimulation, and (2) to investigate how SA enhances triterpene biosynthesis by modulating key genes in the mevalonate pathway. By analyzing SA-induced triterpene production and gene expression, this research demonstrates the connection between practical applications and fundamental molecular insights.

## 2. Results

### 2.1. Evaluation of the Effect of SA Concentration on Mycelial Biomass and Triterpenoid Production in TMB

**Figure 2:**
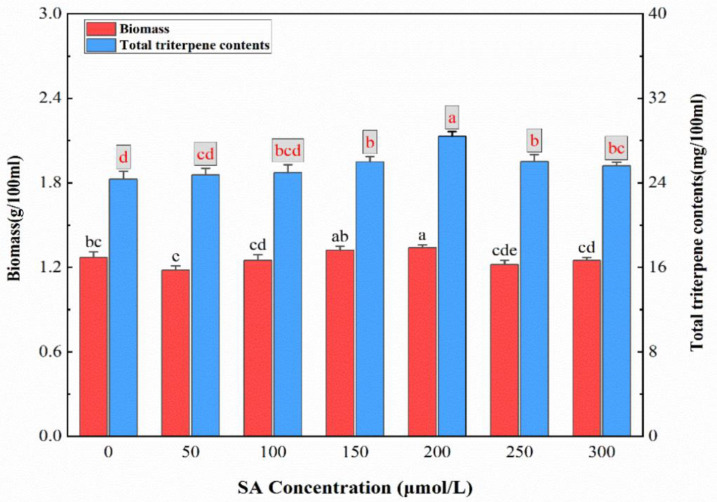
The effects of different concentrations of salicylic acid on the fermentation of TMB. The biomass and the triterpene components were quantitatively analyzed. Salicylic acid was not added to the control. All fermentations were carried out at 24 °C for 7 days. All data were in triplicate (mean ± standard deviation). Different letters indicate significant differences (*p* < 0.05, according to the Duncan test).

The total triterpene components were treated with different concentrations of SA, as shown in Figure 2. For SA concentrations between 50 μmol/L and 200 μmol/L, whenever the SA concentration increased, the total triterpene components also increased gradually, but the mycelium biomass did not increase significantly. At a concentration of 200 μmol/L SA, the total triterpene components reached their peak, which was 1.21 times higher than the control, indicating that this SA concentration induces the most significant increase in total triterpene production. Furthermore, the mycelium biomass also achieved its highest level. Moreover, raising the SA concentration from 200 μmol/L to 300 μmol/L initiated a gradual decrease in the total triterpene components and a decline in mycelium biomass below the control level, signifying that high concentrations of SA are detrimental to cell growth.

### 2.2. Influence of Cultivation Duration on Mycelial Biomass and Total Triterpenoid Content of TMB

**Figure 3:**
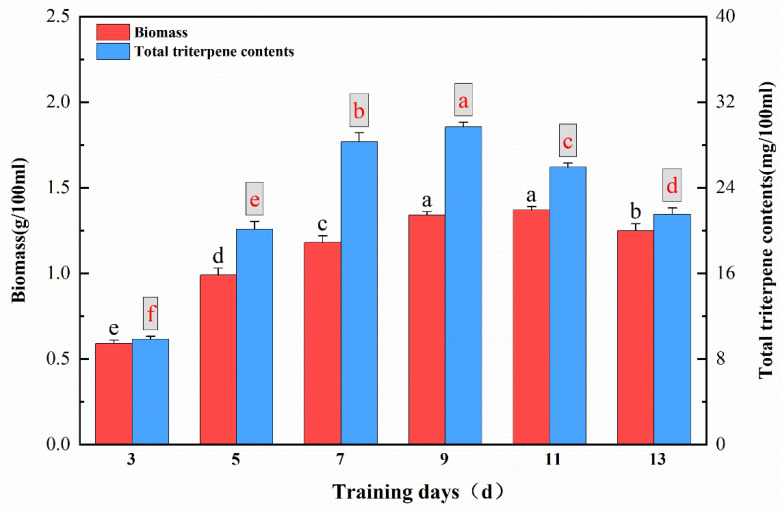
Fermentation days. The effect of 200 µmol/L salicylic acid on the fermentation of TMB. The biomass and the components of triterpenes were quantitatively analyzed. All data were in triplicate (mean ± SD). Different letters indicate significant differences (*p* < 0.05, according to the Duncan test).

200 µmol/L SA was added on the 0th day. The total triterpene components and mycelium biomass in different periods under SA treatment showed that with an increase in days spent in culture, the components of total triterpenes increased rapidly from the 3rd day to the 7th day, and the mycelium biomass changed synchronously with the trend of total triterpene synthesis. The total triterpene components increased slowly from the 7th day to the 9th day and reached their peak on the 9th day, while the mycelium biomass reached its peak on the 11th day. With longer times in culture, the components of total triterpenes and mycelium biomass began to decrease dramatically (Figure 3).

### 2.3. Expression of Related Genes Under Different Concentrations of Salicylic Acid

**Figure 4:**
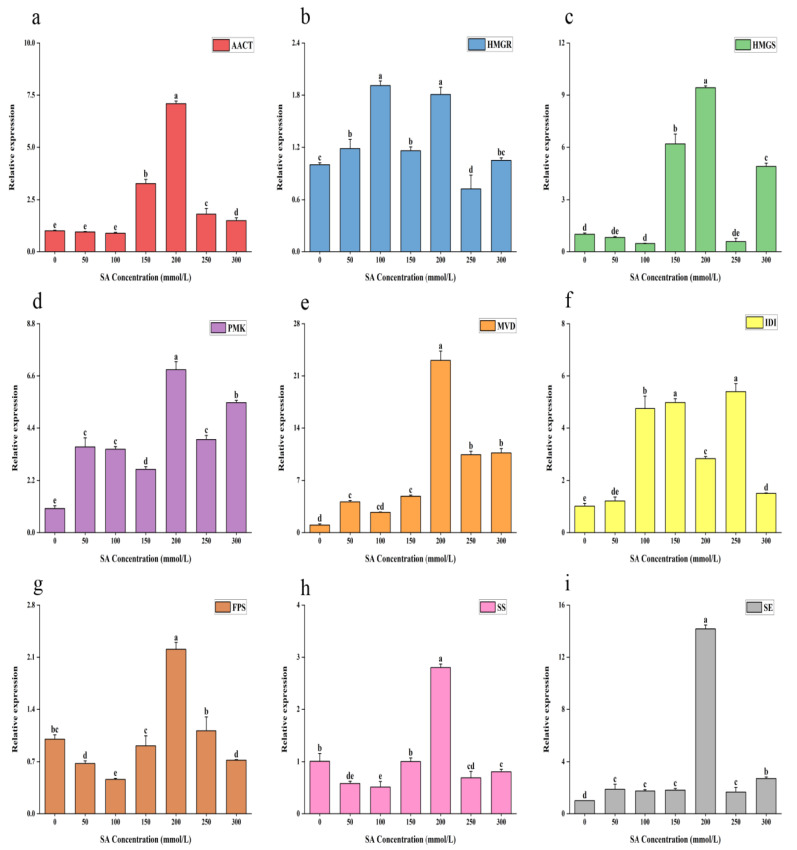
Relative expression of genes in the MVA pathway of TMB treated with salicylic acid at different concentrations. (**a**) Transcription levels of *AACT*. (**b**) Transcription levels of *HMGR*. (**c**) Transcription levels of *HMGS*. (**d**) Transcription levels of *PMK*. (**e**) Transcription levels of *MVD*. (**f**) Transcription levels of *IDI*. (**g**) Transcription levels of *FPS*. (**h**) Transcription levels of *SS*. (**i**) Transcription levels of *SE*. All fermentations were carried out at 24 °C for 7 days. All data were in triplicate (mean ± SD). Different letters indicate significant differences (*p* < 0.05, according to the Duncan test).

The treatment of TMB with SA dramatically increased the components of total triterpenes, a result that is most likely achieved by increasing the expression of related genes in the triterpene biosynthesis pathway. In order to further discuss the effects of different concentrations of SA on the synthesis of total triterpenes, the expression of related genes was studied (Figure 4). Most of the genes were upregulated after SA induction. Moreover, under the optimal concentration of SA for maximum total triterpene production, the transcriptional levels of several genes were at their highest, which was consistent with the changing trend of total triterpene components stimulated by diverse concentrations of SA. For example, the transcriptional levels of *AACT*, *SS*, *MVD*, and *FPS* were highest at 200 μmol/L, at which they were, respectively, 7.09, 2.8, 23, and 2.2 times higher than in the control samples. However, there were some genes whose highest expression concentrations were not 200 μmol/L. The expression of *HMGR* was highest at 100 μmol/L, at which level it was 1.9 times that of the control sample, and the expression of *IDI* was highest at 250 μmol/L, at which it was 5.4 times that of the control sample.

### 2.4. Analysis of Correlation Between Total Triterpenes and Genes Under Salicylic Acid Treatment

In order to further study the correlation between the total triterpene components, mycelium biomass, and transcription level of several triterpene biosynthesis genes under SA treatment, Spearman correlation analysis (OriginLab Corporation, Northampton, MA, USA) was undertaken. The results demonstrated that there was a very significant positive correlation between the total triterpene components and mycelial biomass, and the correlation coefficient was 0.64 (*p* < 0.01). The analysis of the total triterpene components and the triterpene biosynthesis gene transcription level showed that the relative transcription levels of *MVD*, *AACT*, *PMK*, and *SE* genes were considerably positively correlated with the total triterpene components, and the correlation coefficients were 0.86, 0.82, 0.72, and 0.72, respectively (*p* < 0.001). Thus, it is suggested that these genes may play a critical role in triterpene biosynthesis.

**Figure 5:**
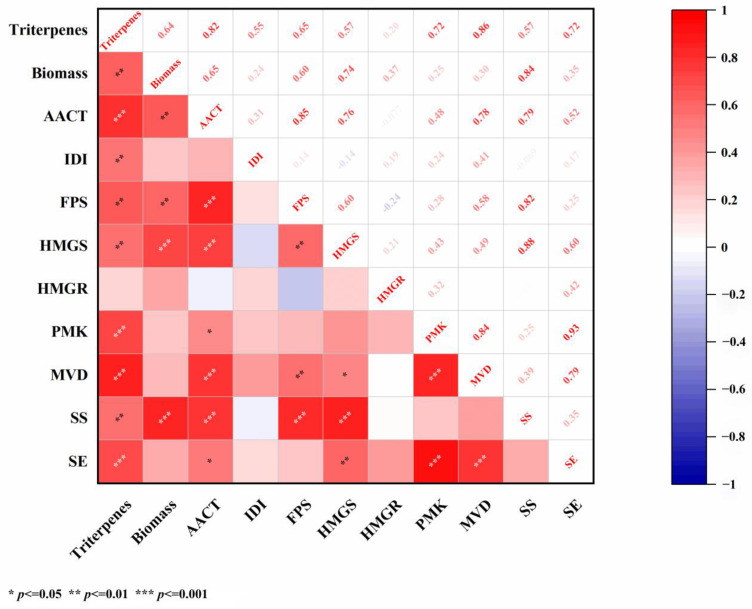
Intergroup correlation analysis of triterpenes, mycelium biomass, and triterpene synthesis genes.

In addition, compared with the four genes substantially related to total triterpene synthesis, the *AACT* gene was significantly positively correlated with the *FPS*, *SS*, *MVD*, and *HMGS* genes; the correlation coefficients were 0.85, 0.79, 0.78, and 0.76, respectively (*p* < 0.001). Similarly, the *MVD* genes were positively correlated with the *PMK* and *SE* genes; the correlation coefficients were 0.84 and 0.79, respectively (*p* < 0.001). There was a fundamental positive correlation between the *PMK* gene and the *SE* gene, and the correlation coefficient was 0.93 (*p* < 0.001) (Figure 5).

### 2.5. Dynamic Change Trend of Key Genes with Time Under Salicylic Acid Treatment

**Figure 6:**
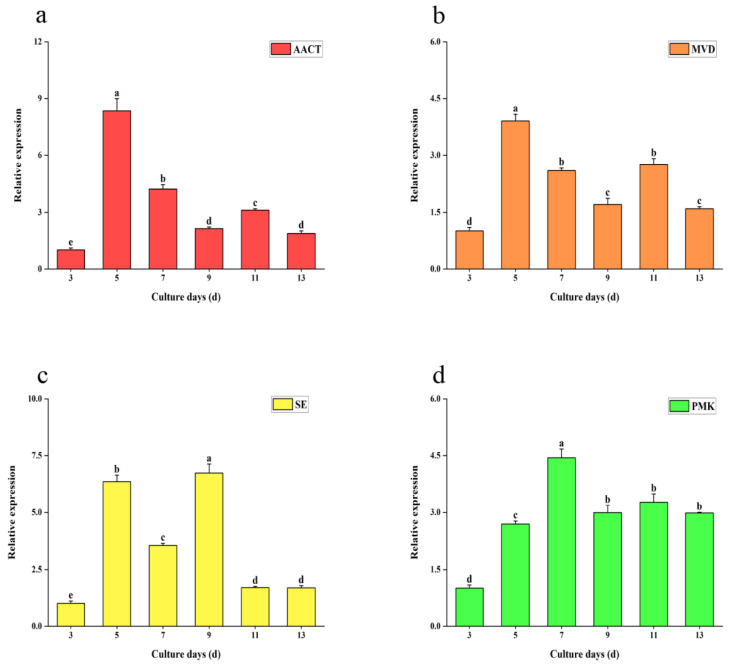
Relative expression of key genes in the MVA pathway in TMB cultured for different days under the treatment of optimal concentrations of salicylic acid. (**a**) Transcription levels of *AACT*. (**b**) Transcription levels of *MVD*. (**c**) Transcription levels of *SE*. (**d**) Transcription levels of *PMK*. All data were in triplicate (mean ± SD). Different letters indicate significant differences (*p* < 0.05, according to the Duncan test).

The genes with a high correlation between metabolite accumulation and gene expression (*p* < 0.001) were selected. The transcription levels of four key genes obtained by correlation analysis were further studied by the qRT-PCR method, and the transcriptional levels of total triterpene biosynthesis genes (*AACT*, *SE*, *MVD*, and *PMK*) were dynamically detected. The results showed that under the stimulation of SA, the highest transcription levels of *AACT* and *MVD* on the 5th day were, respectively, 8.3 times and 3.9 times that of the control group; the highest *PMK* transcriptional level on the 7th day was about 4.4 times that of the control group; and the highest *SE* transcriptional level on the 9th day was about 6.7 times that of the control group (Figure 6).

### 2.6. Cluster Expression Analysis of Related Genes in Response to Salicylic Acid in Different Periods

**Figure 7:**
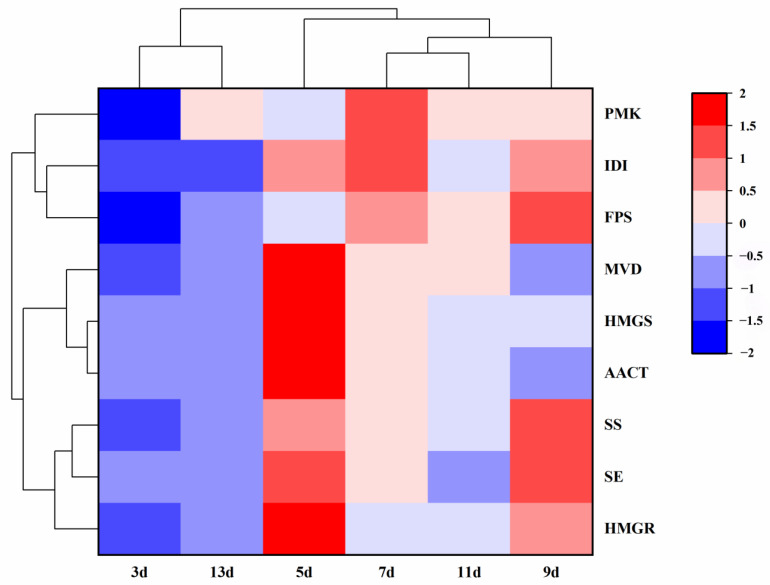
A cluster analysis heat map of salicylic acid’s effect on gene changes in the pathway for different days spent in culture.

The transcriptional data of related genes in different periods stimulated by SA were obtained by qRT-PCR. A cluster analysis was carried out, and a cluster heat map was drawn. The results showed that the responses of the three crucial clusters—3d and 13d, 5d and 7d, and 9d and 11d—changed gradually with time, and the expression was high at 5, 7, and 9 days. Three days in the early stage of fermentation and thirteen days in the later stage of fermentation were low-expression, which was also consistent with the synthesis of total triterpenes (Figure 7).

Additionally, some biosynthetic genes with similar expression patterns were clustered together, and the clustering of similar gene expression revealed three main clusters: The expression patterns of *AACT* and *HMGS*—upstream genes of the triterpene pathway—were closely clustered into a group. The expression patterns of *IDI* and *FPS*—which are in the upper reaches of the triterpene metabolic pathway—were clustered into a cluster. The expression patterns of *SE* and *SS*—both downstream genes—were closely clustered into a cluster. The outcomes of gene clustering were compatible with the positions of genes in the triterpene synthesis pathway, which verified the reliability of the results of cluster analysis.

## 3. Discussion

Triterpenes are the principal active compounds in medicinal and edible fungi [22,23]. SA has extensive physiological effects and plays a vital role in regulating a variety of physiological processes, including the plant immune response [24]. The role of SA in fungi primarily involves stimulating the accumulation of secondary metabolites, such as triterpenoids, by modulating key metabolic pathways and regulating fungal growth [25,26,27]. Based on these findings, SA was applied to stimulate the production of total triterpenoids and mycelial biomass in TMB and to assess its effects on gene expression, particularly for genes involved in secondary metabolite biosynthesis.

In a given culture system, the inducer dose and fermentation time significantly influence metabolite production and cell growth [28,29]. The biosynthesis of total triterpenes in *Ganoderma lucidum* was enhanced by SA, with the optimal concentration for triterpene production found to be 233 μmol/L [30]. This finding provides a valuable reference point for our investigation into the effects of SA on TMB, offering insights into the optimal conditions for triterpenoid biosynthesis in this species. In contrast, *Phellinus igniarius* mycelia exhibited the highest content of terpenoids after a 4-day treatment with SA at a concentration of 350 μmol/L [13]. In our current investigation, TMB was treated with 0–300 μmol/L SA, and the optimal conditions for triterpenoid biosynthesis and mycelial biomass were determined to be when TMB was cultured for 9 days at 200 μmol/L SA. The optimal SA concentration and fermentation time vary among different fungal species, and no research has yet definitively shown whether SA can effectively promote triterpenoid production in TMB. This study provides new insights into the enhancement of triterpenoid biosynthesis in TMB using SA.

TMB exhibits multiple advantages over plants and other fungi in triterpenoid biosynthesis, including a reduced growth cycle, less complex cultivation requirements, and enhanced genetic tractability. These factors collectively contribute to its improved economic feasibility, particularly for large-scale industrial production. Furthermore, TMB synthesizes a broad spectrum of triterpenoids with unique biological activities, positioning it as a promising candidate for pharmaceutical and cosmetic applications. Through salicylic acid induction experiments, this study furnishes the first empirical evidence supporting TMB’s potential for augmented triterpenoid biosynthesis, thereby underscoring its relevance for these sectors. The induction by SA notably elevated the triterpenoid yield, offering an innovative strategy to optimize fungal biosynthetic product output. In comparison to plants and other basidiomycetes, TMB exhibits superior efficiency and cost-effectiveness in triterpenoid production, presenting significant biotechnological advantages. Therefore, TMB holds significant promise as an optimal microbial platform for industrial-scale triterpenoid production, meriting further exploration in biotechnological applications.

Salicylic acid, a subject of significant academic interest and extensive investigation in recent literature, plays a pivotal role in both plants and fungi. It has been demonstrated that SA facilitates the biosynthesis of secondary metabolites in plants and triterpenoids in fungi through intricate gene regulatory mechanisms. For instance, the application of SA to *Tanacetum parthenium* leaves leads to a substantial increase in microalactone concentrations and modulates key genes involved in the early stages of the biosynthetic pathway, such as *HMGR* and *DXR* [31]. In plants like ginseng, exogenous SA treatment has been shown to regulate genes involved in triterpenoid biosynthesis [32]. Recent studies in fungi have shown a significant increase in triterpenoid production following SA treatment. Specifically, the biosynthesis of squalene-related genes plays a key role in the MF + SA induced elevation of triterpenoid content in *Morchella eximia* [33]. These findings provide valuable insights into the molecular mechanisms of triterpenoid biosynthesis in both plants and fungi, contributing directly to the advancement of biotechnological applications. However, further research is needed to fully assess the effects of SA across various fungal species. Moreover, its role in advancing metabolic engineering strategies remains to be explored. Given TMB’s high production efficiency and low cultivation costs, SA presents a promising approach to enhancing triterpene yield and optimizing overall production efficiency.

For a long time, the biosynthesis of terpenes has been considered to be derived from acetyl-CoA through the mevalonate pathway, which is therefore referred to as the MVA pathway [34]. Given the effect of exogenous chemicals on the expression of genes involved in triterpenoid biosynthesis in fungi [35], we selected key genes known to play crucial roles in this pathway for analysis. In this research, we performed qRT-PCR on TMB stimulated by differential concentrations of SA, using the genes involved in the triterpene pathway as the subjects of investigation. The results showed that *AACT*, *MVD*, and *SE* genes involved in triterpenoid biosynthesis were significantly upregulated in response to SA at different concentrations, with most genes showing effective upregulation. Especially when stimulated with 200 μmol/L SA, the transcriptional levels of *AACT*, *HMGS*, *HMGR*, *PMK*, *MVD*, *FPS*, *IDI*, *SE*, and *SS* were higher compared to those in the untreated group. Thus, we hypothesize that SA-induced triterpene biosynthesis in TMB is closely linked to gene expression in the MVA pathway.

The regulation of terpenoid biosynthesis is largely driven by the expression of key genes in the mevalonate pathway, which is crucial for the production of terpenoid precursors [36]. Specifically, it has been previously described in the literature that *AACT* and *SE* are the quintessential structural genes for triterpene synthesis in jujube [37]. Another study illustrated that the increase in ginsenoside contents in *Panax quinquefolium* plants, induced by exogenous substances, may be associated with the regulation of decisive genes such as *MVD*, *PMK*, *DXP*, and others. To identify the genes most closely associated with total triterpene synthesis, we analyzed changes in gene transcription levels, metabolite composition, and mycelium biomass following SA treatment. By creating a correlation heatmap, we discovered that four genes (*AACT*, *MVD*, *PMK*, and *SE*) exhibited momentous correlations with changes in triterpene components (Table 1). After identifying the four pivotal genes, their transcription levels were evaluated at different time points following SA stimulation. These findings revealed that the expression patterns of these genes varied in response to SA induction at different developmental stages. This finding is consistent with previous research, suggesting that gene expression demonstrates temporal specificity [29]. These results provide compelling evidence that exogenous chemicals can modulate the expression of key genes in the terpenoid biosynthesis pathway, leading to increased triterpene production. In this research, we conducted the first molecular-level relative quantitative analysis of TMB triterpene pathway synthesis genes. Furthermore, we provided experimental data for the first time on four genes (*AACT*, *MVD*, *PMK,* and *SE*) substantially related to total triterpene synthesis in TMB induced by SA through gene-metabolic correlation analysis. This information is valuable for further elucidating the regulatory mechanisms underlying triterpene biosynthesis and for identifying potential targets for metabolic engineering to enhance triterpene production

Previously reported outcomes indicated that abiotic stress affects the function of the SmWRKY family in the herb *Salvia miltiorrhiza*, influencing secondary metabolite accumulation, and that biosynthetic genes with similar expression patterns are grouped together [38]. In this study, heatmap and clustering analyses were employed to assess the transcriptional levels of genes derived from the TMB culture process. The results revealed that the 3d/13d, 7d/9d, and 11d samples clustered together, and the *IDI*/*FPS*, *SE*/*SS*, and *AACT*/*HMGR* genes formed a distinct cluster. These findings suggest that genes activated by SA in the TMB triterpene synthesis pathway, which exhibit similar expression patterns, may share comparable functional roles. This research represents the first attempt to perform cluster analysis on the spatio-temporal expression levels of TMB genes, offering insights into the molecular mechanisms underlying gene function in the triterpene synthesis pathway and their contribution to secondary metabolite accumulation. Although we inferred the genes’ role in triterpenoid synthesis through correlation data, direct evidence of causal relationships remains absent. Future studies will include functional assays, such as gene knockout or overexpression, to elucidate their roles in the pathway and further support our conclusions.

## 4. Materials and Methods

### 4.1. Strains of Athelia Termitophila and Their Cultural Conditions

The *A. termitophila* was obtained by our research team at Northwest University, Xi’an. Information on biological preservation is available in CCTCC M 2018446. The strains of TMB were inoculated into Comprehensive Potato Solid Medium (CPDA) and stored in a 4 °C incubator. Prior to use, the original strain was inoculated into fresh CPDA and cultured in a 24 °C incubator for 7 days. Five agar blocks of activated TMB were inoculated into Comprehensive Potato Liquid Medium (CPDB) with a perforator, and the first-class seed liquid was prepared at 24 °C, 160 r, and 7 days.

### 4.2. Composition of the Medium

Comprehensive Potato Solid Medium (CPDA) consists of the following: potato extract powder (24 g), potassium dihydrogen phosphate (3 g), magnesium sulfate (1.5 g), and agar (20 g). Comprehensive Potato Liquid Medium (CPDB) consists of the following: potato extract powder (24 g), potassium dihydrogen phosphate (3 g), and magnesium sulfate (1.5 g). Sucrose liquid medium consists of the following: sucrose (30 g), yeast powder (6 g), and potassium dihydrogen phosphate (3 g).

### 4.3. Method for the Determination of Mycelial Biomass

The TMB mycelium, which had been cultured in a shaking flask, was transferred to a Brinell funnel equipped with four layers of gauze. During the filtration process, the sample underwent thorough rinsing with distilled water before being subjected to vacuum filtration using a pump. Subsequently, it was dried in a blast drying box at 50 °C until reaching constant weight, which served as an indicator of mycelium biomass. Each independent experiment was repeated three times.

### 4.4. Extraction, Detection, and Analysis of Total Triterpenes in a Mycelium

The dried TMB mycelium was pulverized into a powder, accurately weighed to 0.5 g, and then placed in a test tube with a glass stopper. An extract with 10 mL of methanol was prepared using ultrasonic conditions at 160 W for 40 min. The mixture was then centrifugally filtered, and the supernatant was recovered and transferred to a 10 mL test tube. Next, methanol was adjusted to the scale, and the remaining filtrate became the sample solution for testing. The determination method was improved based on previous studies [39]. A total of 0.2 mL of oleanolic acid reference solution, sample solution, and distilled water were taken separately and placed in three different plug test tubes with a capacity of 10 mL each. After steaming in a water bath at 100 °C, 0.2 mL of a vanillin-glacial acetic acid solution (5%) was added to each tube and shaken well. Then, a perchloric acid solution (0.8 mL) was added, and the tubes were shaken thoroughly. The tubes were heated for 15 min in a water bath at 70 °C before being immediately cooled for 5 min in an ice water bath. Then, glacial acetic acid was added to adjust the volume to 5 mL, and the tubes were shaken well. The chromogenic solution of the reference substance, the chromogenic solution of the sample to be tested, and the blank control chromogenic solution were obtained. A spectrophotometer measured absorbance at a wavelength of 548 nm using the blank chromogenic solution as a reference point. The standard curve of total triterpenes served as a reference for calculating the components of triterpenes.

### 4.5. Optimization of Salicylic Acid Concentration for the Total Triterpenoid Synthesis of TMB

SA was dissolved using ethanol as the solvent, and the bacteria were removed using a 0.45 mm nylon filter membrane. The SA solution was added to the culture at the beginning of the experiment so that the final concentrations of SA were 0, 50, 100, 150, 200, 250, and 300 μmol/L. The control group was treated with the same volume of ethanol. All the experiments were carried out in triplicate, and the values are shown as the average ± standard deviation.

### 4.6. Effects of Different Incubation Periods on the Yield of Total Triterpenoids in TMB

SA was dissolved in ethanol and filtered through a 0.45 mm nylon membrane to remove bacteria. This filtered SA solution was added to the culture at the beginning of the experiments, on the 0th day. The cells were cultured according to the different culture days (3, 5, 7, 9, 11, and 13 days). All experiments were carried out in triplicate, and the values are shown as the average ± standard deviation of total triterpenes.

### 4.7. Measurement of the Gene Transcription Level by qRT-PCR

The mycelia treated with SA were collected, the total RNA was extracted from the mycelia, and the first-strand cDNA was reverse-transcribed into first-strand cDNA using the EvoM-MLV Reverse Transcription Premixed Kit (Accurate Biotechnology (Hunan) Co., Ltd. Changsha, Hunan Province, China). The cDNA was then considered to be the template for qRT-PCR determination. Onward, the Premix Pro Taq HS qPCR Kit (Accurate Biotechnology (Hunan) Co., Ltd. Changsha, Hunan Province, China) was used for RT-PCR. The gene sequence was screened from transcriptome data, and then the primers were designed using Primer 5 (Table 2). The relative expression of each gene was evaluated by gene-specific primers and standardized by the TMB 18S rRNA gene, which was found to be stable under our experimental conditions. For each gene, the expression level of the reference sample was 1.0, and the results of the samples under other conditions were expressed as the multiple changes of the mRNA level relative to the reference sample. Furthermore, the qRT-PCR reaction was carried out in the Line-Gene 9660 Plus Real Time Fluorescence Quantitative PCR Detection System (Bioer Technology, Hangzhou, Zhejiang Province, China). The program was carried out according to the instructions and calculated using the 2^−ΔΔCt^ method.

### 4.8. Statistical Analysis

All the experiments were independently repeated three times, and the data were analyzed using IBM SPSS Statistics (Version 26.0, Armonk, NY, USA: IBM Corp.) to obtain mean ± standard deviation (SD). Univariate analysis and the Duncan post-test were used for significance analysis. Origin Pro 2022 (OriginLab Corporation, Northampton, MA, USA) was employed for correlation analysis, cluster analysis, and drawing.

## 5. Conclusions

To summarize, this study highlights the dual significance of SA in promoting triterpene production and elucidates the molecular mechanisms underlying this process in TMB. We successfully determined the optimal concentration of SA that promotes the production of total triterpenes. Additionally, we identified the corresponding duration spent in culture that led to the highest total triterpene production in the TMB. In addition, the results of this research illustrate that SA stimulates the biosynthesis of total triterpenes by inducing gene transcription of key enzymes in the triterpene synthesis pathway (*AACT*, *HMGS*, *HMGR*, *PMK*, *MVD*, *FPS*, *IDI*, *SE*, and *SS*). Through correlation analysis and dynamic spatio-temporal transcriptional profiling, we identified four genes—*AACT*, *MVD*, *PMK*, and *SE*—as being most strongly associated with total triterpene synthesis. Finally, we assessed the spatio-temporal expression levels of the key genes in TMB using cluster integration. These findings not only advance our understanding of the molecular regulation of triterpene synthesis but also provide a foundation for future metabolic engineering efforts to optimize triterpene production in TMB.

## Figures and Tables

**Table 1:** An analysis of the correlation between total triterpenes, key genes, and mycelium biomass.

Project	Correlation Coefficient				
	Biomass	*AACT*	*SE*	*MVD*	*PMK*
Triterpenes	0.64 **	0.82 ***	0.72 ***	0.86 ***	0.72 ***
Biomass	1	0.65 **	0.35	0.3	0.25

*p* < 0.01 **, *p* < 0.001 ***.

**Table 2:** Primer synthesis sequence.

Gene Abbreviation	Gene Full Name	Primer Name and Size	Sequence (5′-3′)
AACT	Acetyl-Coa Acetyltransferase	AACT-221-F	CTATCAAGGGCAAGAAGGGTG
		AACT-221-R	GGAGATGACTTTGGCAAGAGGTT
HMGS	3-Hydroxy-3-Methylglutaryl-Coa Synthase	HMGS-153-F	GTCTTCGATGGAGTGTCTAAGGG
		HMGS-153-R	ACCGATAGATTTTGGGTCGATGT
HMGR	3-Hydroxy-3-Methylglutaryl-Coa Reductase	HMGR-127-F	ACCAACCAACAACGGCGA
		HMGR-127-R	GCCCTTCACACCGAGCAT
PMK	Phosphomevalonate Kinase	PMK-200-F	CGGATGATGCTTGCTGATGT
		PMK-200-R	GCTGTGAGCGTGTAAGTGCC
MVD	Mevalonate Diphosphate Decarboxylase	MVD-191-F	TATGGTTGAACGGCAAGGTAGA
		MVD-191-R	TGATGCGGAAGATGCGAGA
IDI	Isopentenyl-Diphosphate Delta Isomerase	IDI-138-F	GTGACACCCAATGAGAACGAA
		IDI-138-R	CCACCAGCCGAACAGGAA
FPS	Farnesyl Diphosphate Synthase	FPS-187-F	CATCGGCAAACAAACTGGCA
		FPS-187-R	GAGAACAAGGAAGGCACCGA
SS	Squalene Synthase	SS-182-F	TGGATACCATTGAGGACGACA
		SS-182-R	TGGAGAAAGGCGGTTGACT
SE	Squalene Epoxidase	SE-132-F	CGGTCGTGCTCGTGAAGGG
		SE-132-R	GGAGGATGTGCGTCGTTATATGC
18S rRNA	18S ribosomal RNA	18S rRNA-164-F	ACATCCGCCATCCATTCC
		18S rRNA-164-R	TCGCTGCCCATCACCATA

## Data Availability

Data will be made available on request.
